# Neutralizing hepatic apolipoprotein E enhances aged bone fracture healing

**DOI:** 10.1038/s41413-025-00489-y

**Published:** 2026-01-22

**Authors:** Mingjian Huang, Abhinav Reddy Balu, Kristin Happ Molitoris, Akshay Bareja, Gurpreet Singh Baht

**Affiliations:** 1 Department of Orthopaedic Surgery, Duke University, Durham, NC USA; 2 Duke Molecular Physiology Institute, Duke University, Durham, NC USA; 3 Feinberg School of Medicine, Northwestern University, Chicago, IL USA; 4 Department of Medicine, Duke University, Durham, NC USA

**Keywords:** Bone, Physiology

## Abstract

Advanced age impairs bone fracture healing; the underlying mechanism of this phenomenon remains unknown. We determined that apolipoprotein E (ApoE) increases with age and causes poor fracture healing. After deletion of hepatic ApoE expression (ΔApoE), 24-month-old ΔApoE mice displayed a 95% reduction in circulating ApoE levels and significantly improved fracture healing. ApoE treatment of aged BMSCs inhibited osteoblast differentiation in tissue culture models; RNA-seq, Western blot, immunofluorescence, and RT-PCR analyses indicated that the Wnt/β-catenin pathway is the target of this inhibition. Indeed, we showed that ApoE had no effect on cultures with stabilized β-catenin levels. Next, we determined that Lrp4 serves as the osteoblast cell surface receptor to ApoE, as expression of Lrp4 is required in ApoE-based inhibition of Wnt/β-catenin signaling and osteoblast differentiation. Importantly, we validated this ApoE-Lrp4-Wnt/β-catenin molecular mechanism in human osteoblast differentiation. Finally, we identified an ApoE-neutralizing antibody (NAb) and used it to treat aged, wildtype mice 3 days after fracture surgery resulting in fracture calluses with 35% more bone deposition. Our work here identifies novel liver-to-bone cross-talk and a noninvasive, translatable therapeutic intervention for aged bone regeneration.

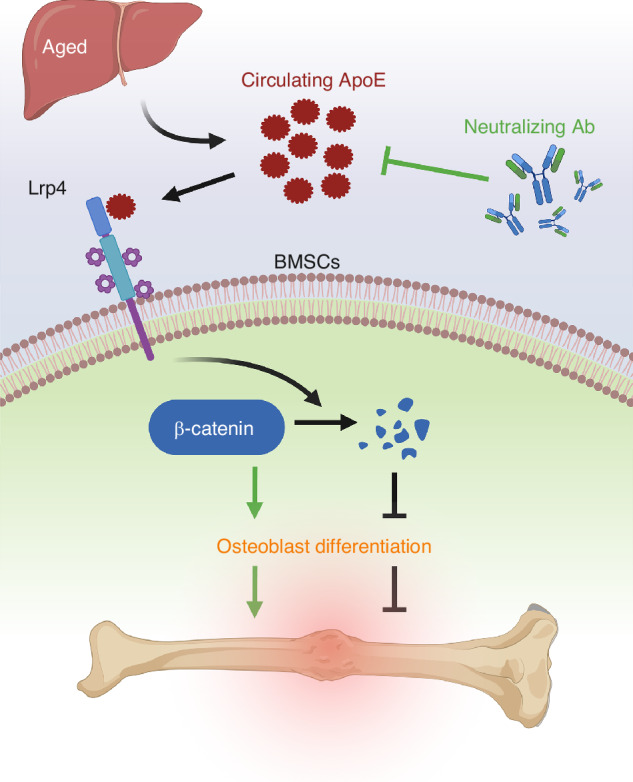

## Introduction

Fracture healing diminishes with advanced age; it is associated with increased morbidity and mortality and often requires invasive surgical interventions resulting in an increased healthcare burden and financial burden on the patient.^1–4^ Current treatment strategies to improve bone healing rely on compounds that must be surgically inserted at the site of injury, a process that further increases the burden on the patient and often leads to complications associated with the invasive surgery itself, and with extended bed rest and a lack of mobility. Unfortunately, success of these compounds is inconsistent and is associated with numerous complications such as an improper graft or fusion, heterotopic bone formation, and various urologic problems.^5–7^ Thus, current strategies to improve aged bone fracture healing fall short of optimal care. The development of non-invasive bone regeneration strategies would improve health outcomes and lower the health burden of orthopedic injuries in advanced age.

We used heterochronic parabiosis models joining the circulatory system of a young mouse to that of an aged mouse to identify endogenous factors that improve aged fracture healing and that could therefore serve as non-invasive injectable treatments. After establishing blood sharing, we determined that fracture healing in aged mice was improved.^8,9^ From this model, we identified ApoE as an aging factor: circulating ApoE levels increase with age in patients and in mice and ApoE inhibits bone fracture healing.^10^ Greater than 90% of circulating ApoE is secreted from hepatocytes of the liver. As such, ApoE potentially represents a humoral factor functioning in organ cross-talk. The molecular mechanism by which ApoE inhibits bone repair is unknown; targeting ApoE in a therapeutic strategy to improve fracture healing in the elderly has never been explored.

Here, we identify the critical role of hepatic ApoE and its underlying mechanism in age-associated shortcomings of bone fracture healing. ApoE alters osteoblast differentiation and activity during fracture healing. We identified the cell surface receptor that binds ApoE as well as the signaling pathway used by ApoE to alter osteoblast biology. Most importantly, we confirmed all of our mechanistic findings in human tissue culture models. Finally, based on these findings, we developed a non-invasive translatable therapeutic intervention to improve aged bone fracture healing.

## Results

### Hepatic ApoE impairs aged bone regeneration

**Fig. 1:**
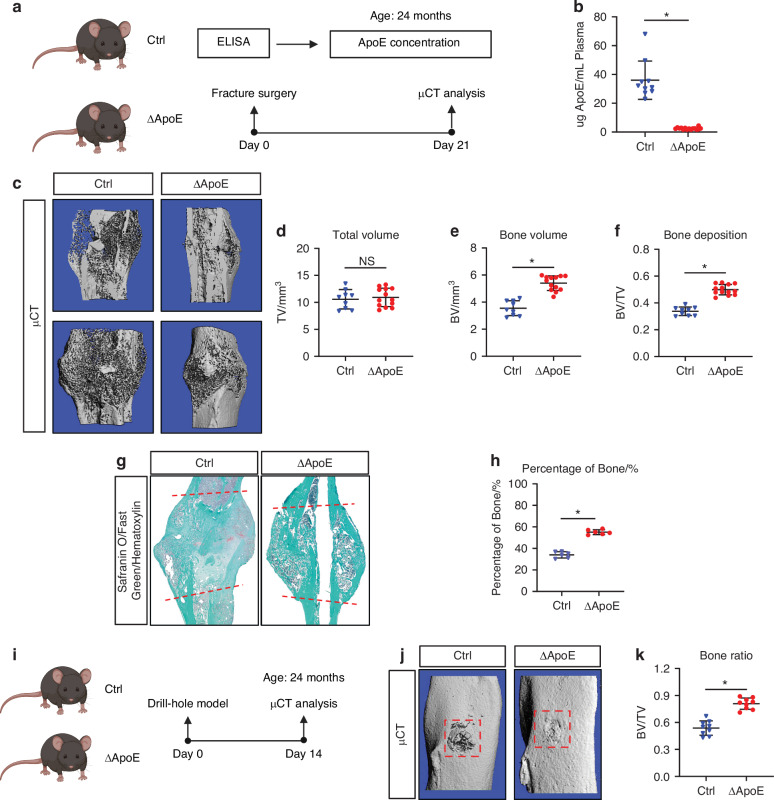
Loss of hepatic ApoE expression increases bone deposition in aged fracture callus. **a** Schematic diagram of the tibia fracture model in 24-month-old control (ApoE^fl/fl^, Ctrl) mice and liver-specific ApoE knockout (ApoE^Alb^, ΔApoE) mice. **b** ELISA was used to measure ApoE concentration in the plasma of mice (Ctrl, *n* = 10; ΔApoE, *n* = 15). **c** 21-day fracture calluses were assessed using micro-CT analysis to determine (**d**) total volume (TV), (**e**) bone volume (BV), and (**f**) bone ratio (BV/TV) (Ctrl, *n* = 9; ΔApoE, *n* = 13). **g** Safranin O/fast green/hematoxylin staining was used to stain the fracture calluses and (**h**) histomorphometric analysis was used to quantify the amount of bone within the fracture callus (Ctrl, *n* = 6; ΔApoE, *n* = 6). **i** Schematic diagram of tibial drill hole defect in 24-month-old control (ApoE^fl/fl^, Ctrl) mice and liver-specific ApoE knockout (ApoE^Alb^, ΔApoE) mice. **j** Healing was assessed 14 days after defect using micro-CT and (**k**) bone ratio (BV/TV) in the defect region was determined (Ctrl, *n* = 9; ΔApoE, *n* = 9). Data are presented as mean ± 95% confidence interval. *P < 0.05

The primary source of circulating ApoE is the liver.^11^ To investigate the effect of circulating ApoE on fracture healing we generated mice in which the ApoE gene was selectively nulled in hepatocytes to test whether a loss of hepatic ApoE can improve aged fracture healing. Albumin-Cre mice were crossed with ApoE^fl/fl^ mice to generate hepatocyte-specific knockouts (*Alb-Cre*^*+/−*^*;ApoE*^*fl/fl*^, referred to as ΔApoE) or littermate controls (*ApoE*^*fl/fl*^, referred to as Ctrl) and aged to 24 months (Fig. 1a). ELISA was used on plasma samples to test circulating ApoE concentrations. ΔApoE mice exhibited greater than 90% decrease in plasma ApoE concentration relative to Ctrl (Fig. 1b). Importantly, micro-CT analysis of intact femurs of 24-month-old ΔApoE and Ctrl mice was conducted to determine whether hepatic ApoE affects skeletal development (Fig. S1a, b). No significant differences were identified in cortical thickness, trabecular space, trabecular thickness, nor trabecular number in ΔApoE mice relative to Ctrl (Fig. S1C–F), indicating hepatic ApoE does not affect skeletal development.

Next, 24-month old mice underwent tibial fracture surgery and healing was measured 21 days post-injury using micro-CT and histological assessments. Micro-CT analysis demonstrated that while there was no difference in fracture callus size (total volume, TV), ΔApoE fracture calluses contained 50% more bone volume and had 50% higher bone ratio relative to Ctrl (Fig. 1c–f). Histological analysis corroborated these findings as Safranin O/fast green/hematoxylin staining of paraffin-embedded sections demonstrated a significant increase in bone tissue deposition in ΔApoE fracture calluses (Fig. 1g, h). Furthermore, osteoblast numbers were significantly increased in 21-day ΔApoE fracture calluses while cartilage template was decreased, and osteoclast activity was unchanged (Fig. S2).

To further assess differential bone healing, we used a tibial defect model. 24-month-old ΔApoE and Ctrl mice underwent tibial drillhole surgery and tissues were collected 14 days post-injury; healing was assessed by micro-CT (Fig. 1i). ΔApoE defects contained more bone tissue than did Ctrl defects (Fig. 1j, k) and osteoblast numbers were significantly increased while osteoclast activity was unchanged (Fig. S3).

Collectively, this work demonstrates that the primary source of circulating ApoE is the liver and that circulating ApoE impairs proper fracture healing with age.

### ApoE impairs osteoblast differentiation while inhibiting the Wnt/β-catenin and Hippo pathways

**Fig. 2:**
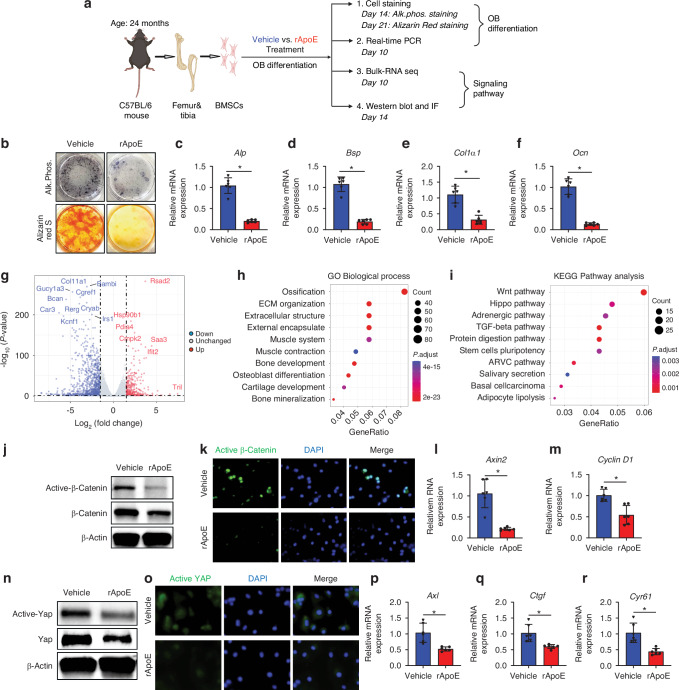
ApoE impairs osteoblast differentiation and inhibits Wnt/β-catenin and Hippo signaling. **a** Schematic diagram of BMSC isolation, culture, subsequent osteogenic differentiation with vehicle or rApoE treatment and subsequent analysis. **b** BMSCs from 24-month-old C57BL/6 mice were cultured and differentiated in osteogenic media containing vehicle or rApoE. Wells were washed, fixed, and stained for Alkaline Phosphatase (Alk. Phos.) or mineral (Alizarin Red) to assess osteoblast differentiation and matrix mineralization, respectively. **c**–**f** Transcripts for osteogenic genes (*Alp*, *Bsp*, *Col1*, and *Ocn*) were measured using RT-PCR (*n* = 6). **g** Bulk RNA-seq was used to identify differentially expressed genes (DEGs) which were presented on a volcano plot using a log_2_(FC)-value of 2 and a *P* value of 0 as cut-offs for DEGs to compare vehicle-treated vs. rApoE-treated groups. **h** Downregulated DEGs were collected for Gene Ontology (GO) biological process analysis and (**i**) KEGG pathway analysis. **j** Western blot analysis of active-${\rm{\beta }}$-catenin and total ${\rm{\beta }}$-catenin protein levels in vehicle-treated and rApoE-treated groups. **k** Immunofluorescence staining (active-${\rm{\beta }}$-catenin) of vehicle- and rApoE-treated osteogenic cultures. **l**, **m** Expression level of$\,{\rm{\beta }}$-catenin target genes *Axin2* and *Cyclin D1* (*n* = 6). **n** Western blot analysis of active-Yap and total-Yap in vehicle-treated and rApoE-treated groups. **o** Immunofluorescence staining (active-Yap) of vehicle- and rApoE-treated osteogenic cultures. **p**–**r** Expression level Yap target genes *Axl*, *Ctgf*, and *Cyr61* (*n* = 6). Data are presented as mean ± 95% confidence interval. *P < 0.05

Here we show that loss of hepatic ApoE expression leads to increased osteoblast number and bone formation within the fracture callus. Thus, our next step was to determine ApoE’s effect on differentiation of aged bone marrow stromal cells (BMSCs) to osteoblasts. BMSCs from 24-month-old wildtype (C57BL/6 J) mice were cultured and differentiated to osteoblasts with vehicle or 100 ng/mL recombinant ApoE (rApoE) (Fig. 2a). Alkaline Phosphatase (Alk. Phos.) staining and Alizarin Red staining demonstrated that rApoE treatment decreased aged osteoblast differentiation and extracellular matrix mineralization (Fig. 2b). Furthermore, rApoE treatment was found to reduce expression of osteogenic marker genes *Alp*, *Bsp*, *Col1α1*, and *Ocn* in RT-PCR analysis (Fig. 2c–f). Interestingly, these findings were recapitulated when using cells from young, 4-month old mice. BMSCs from 4-month old mice were differentiated in osteogenic media containing vehicle or ApoE. As with aged cells, ApoE treatment inhibited osteoblastic differentiation of young cells (Fig. S4).

We performed RNA sequencing (RNA-seq) on aged, ApoE-treated or untreated osteogenic cultures to identify the underlying mechanism of ApoE inhibition of osteoblast differentiation. Differentially expressed genes (DEGs) were depicted using a volcano plot (Fig. 2g) and biological functions of up- and down-regulated DEGs were assessed using gene ontology (GO) analysis. GO analysis indicated that the downregulated DEGs were mainly enriched in ossification, bone development, osteoblast differentiation and bone mineralization (Fig. 2h). We performed Kyoto Encyclopedia of Genes and Genomes (KEGG) analysis of downregulated DEGs and enrichment analysis of KEGG revealed that the downregulated DEGs were mainly involved in the Wnt/β-catenin and the Hippo signaling pathways (Fig. 2i).

Gene set enrichment analysis (GSEA) demonstrated that the Wnt/β-catenin and the Hippo signaling pathways were significantly enriched (*P* < 0.01), a point that was further corroborated by heatmap visualization (Fig. S5). To validate modulation of these pathways, aged BMSCs were cultured in the absence or presence of ApoE and tested using biochemical techniques.

In the Wnt/β-catenin pathway, signaling is dependent on activation of β-catenin, its translocation to the nucleus, and subsequent expression of its transcriptional targets. Western blot analysis demonstrated that active β-catenin and total β-catenin protein levels decreased in response to ApoE treatment (Fig. 2j). Similarly, decreased active-β-catenin immunofluorescence staining was seen in cultures treated with ApoE (Fig. 2k) and Wnt/β-catenin pathway transcription targets, *Axin2* and *Cyclin D1*, were down-regulated by ApoE treatment (Fig. 2l, m).

In the Hippo pathway, signaling is dependent on activation and subsequent nuclear translocation of the Yap/Taz protein complex into the nucleus to induce the TEAD-mediated gene transcription.^12^ Heatmap analysis of Hippo signaling pathway target genes indicated inhibition of this pathway (Fig. S5). Active-Yap and total-Yap protein levels decreased in response to ApoE treatment, as indicated by Western blot analysis and immunoblot staining (Fig. 2n, o). Transcriptional targets of the pathway (*Axl*, *Cyr61*, and *Ctgf*) were also found to be inhibited by ApoE treatment (Fig. 2p–r).

Collectively these findings indicate the involvement of the Wnt/β-catenin and/or Hippo pathways in ApoE-based inhibition of osteoblast differentiation.

### Modulation of Wnt/β-catenin but not Hippo signaling is required for ApoE-based inhibition of osteoblast differentiation

**Fig. 3:**
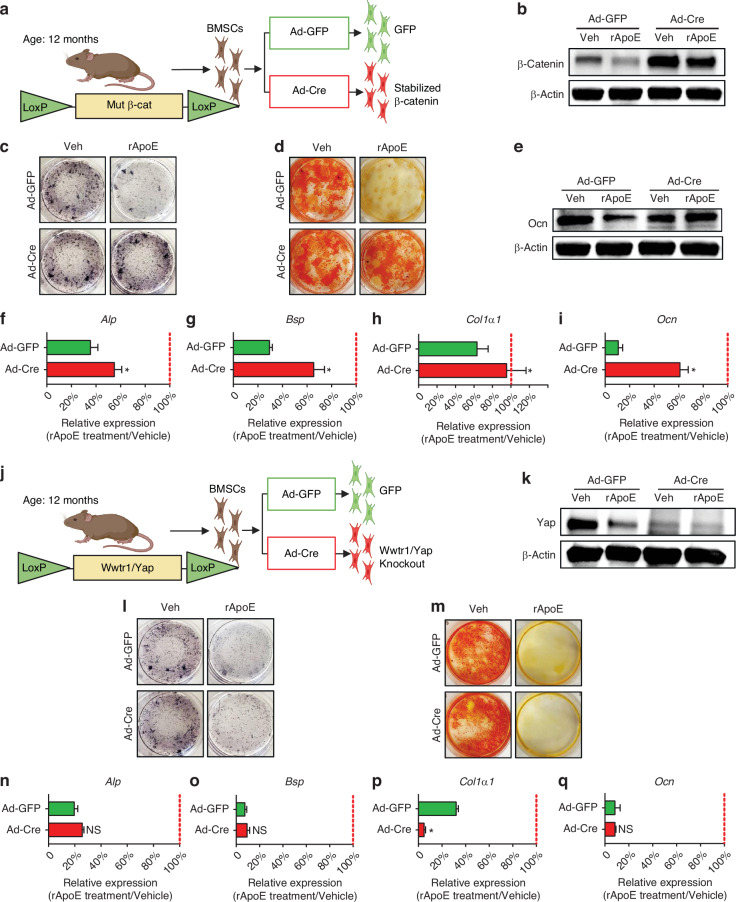
Modulation of the Wnt/${\rm{\beta }}$-catenin but not the Hippo pathway is required for ApoE’s inhibitory effect on osteoblast differentiation. **a** Schematic diagram of experimental design. BMSCs from mutant ${\rm{\beta }}$-catenin loxP mice were cultured and treated with adenovirus carrying Cre-recombinase to generate ${\rm{\beta }}$-catenin stabilized BMSCs (or carrying GFP as control). Cultures then underwent osteogenic differentiation in the presence of vehicle or rApoE. **b** Western blot analysis confirmed stabilization ${\rm{\beta }}$-catenin in BMSCs upon treatment with adenovirus-Cre recombinase. **c** Cells were washed, fixed, and stained for Alkaline Phosphatase or (**d**) mineral (Alizarin Red) to assess osteoblast differentiation. **e** Western blot was used to assess the Col1α1 and OCN protein levels of osteogenic cultures. **f**–**i** Relative expression of osteogenic transcripts (*Alp*, *Bsp*, *Col1a1*, and *Ocn*) was measured in all culture conditions. Transcript expression in rApoE-treated cultures was related transcript expression of vehicle-treated cultures and plotted as Ad-GFP vs Ad-Cre. **j** Schematic diagram of experimental design. BMSCs from mutant Yap/Taz loxP mice were cultured and treated with adenovirus carrying Cre-recombinase to generate Yap/Taz knockout BMSCs (or carrying GFP as control). Cultures then underwent osteogenic differentiation in the presence of vehicle or rApoE. **k** Western blot analysis confirmed loss of Yap expression in BMSCs upon treatment with adenovirus-Cre recombinase. **l** Cells were washed, fixed, and stained for Alkaline Phosphatase or (**m**) mineral (Alizarin Red) to assess osteoblast differentiation. **n** Western blot was used to assess the Col1α1 and OCN protein levels of osteogenic cultures. **o**–**r** Relative expression of osteogenic transcripts (*Alp*, *Bsp*, *Col1a1*, and *Ocn*) was measured in all culture conditions. Transcript expression in rApoE-treated cultures was related transcript expression of vehicle-treated cultures and plotted as Ad-GFP vs Ad-Cre. Data are presented as mean ± 95% confidence interval. *P < 0.05

We found both Wnt/β-catenin and Hippo signaling to be inhibited in concert with ApoE-based inhibition of osteoblast differentiation. To determine whether ApoE functions through the Wnt/β-catenin pathway, we cultured BMSCs from β-catenin-stabilized mice in which Cre-recombinase-based excision results in the production of a stabilized form of β-catenin protein. We chose this model over the loss-of-function β-catenin model because the loss-of-function model itself acts as a strong inhibitory model for osteoblast differentiation. Thus, interpretation of results assessing ApoE inhibitory activity layered on top of the loss-of-function inhibitory activity would be difficult. BMSC cultures from β-catenin stabilized mice were treated with adenovirus encoding green fluorescent protein (Ad-GFP, control) or adenovirus encoding a GFP-labelled Cre-recombinase (Ad-Cre) and were then differentiated in osteogenic media containing either vehicle or rApoE (Fig. 3a). Western blot analysis confirmed that ApoE-treatment lowered β-catenin levels in control samples but could not alter β-catenin levels in Ad-Cre cultures (Fig. 3b). Osteogenic differentiation was assessed by staining cultures, assessing type I collagen and osteocalcin levels, and quantifying osteogenic transcripts. ApoE treatment decreased staining in Ad-GFP control cultures but did not alter staining in Ad-Cre cultures for Alkaline Phosphatase (Fig. 3c) nor for Alizarin Red (Fig. 3d). Osteocalcin (OCN), a marker of osteoblast differentiation, was decreased by ApoE treatment in control cultures but did not change in Ad-Cre cultures (Fig. 3e). Finally, osteogenic transcripts (*Alp*, *Bsp*, *Col1a1*, *Ocn*) from rApoE-treated cultures were measured and related to osteogenic transcripts from vehicle-treated cultures. Ad-Cre-treated cultures were protected from ApoE-based inhibition while Ad-GFP were not (Fig. 3f–i).

To determine whether ApoE functions through the Hippo pathway, we cultured BMSCs from Taz-floxed/Yap-floxed mice in which Cre-recombinase-based excision results in a loss of expression of the Yap and Taz proteins, muting transcriptional activity of the Hippo pathway. Similar to the tissue culture experiment above, cultures were treated with either adenovirus encoding green fluorescent protein (Ad-GFP, control) or adenovirus encoding a GFP-labelled Cre-recombinase (Ad-Cre) and were then differentiated in osteogenic media containing either vehicle or rApoE (Fig. 3j). Western blot analysis confirmed that ApoE-treatment significantly lowered Yap protein levels in control samples but could not alter Yap protein levels in Ad-Cre cultures (Fig. 3k). ApoE treatment decreased staining in Ad-GFP control cultures and likewise in Ad-Cre cultures for Alkaline Phosphatase (Fig. 3l) and for Alizarin Red (Fig. 3m). Furthermore, knockdown of Yap/Taz protein expression did not affect sensitivity to ApoE treatment. Both Ad-Cre and Ad-GFP treated cultures displayed similar levels of inhibition from ApoE as depicted by measurement of osteogenic transcripts (*Alp*, *Bsp*, *Col1α1*, *Ocn*) (Fig. 3n-q).

Collectively, these data indicate that ApoE-treatment of osteoblasts inhibits both the Wnt/β-catenin and Hippo pathways; however, ApoE’s osteoblast inhibitory activity functions through the Wnt/β-catenin pathway.

### Lrp4 serves as the osteoblast cell-surface receptor for ApoE

**Fig. 4:**
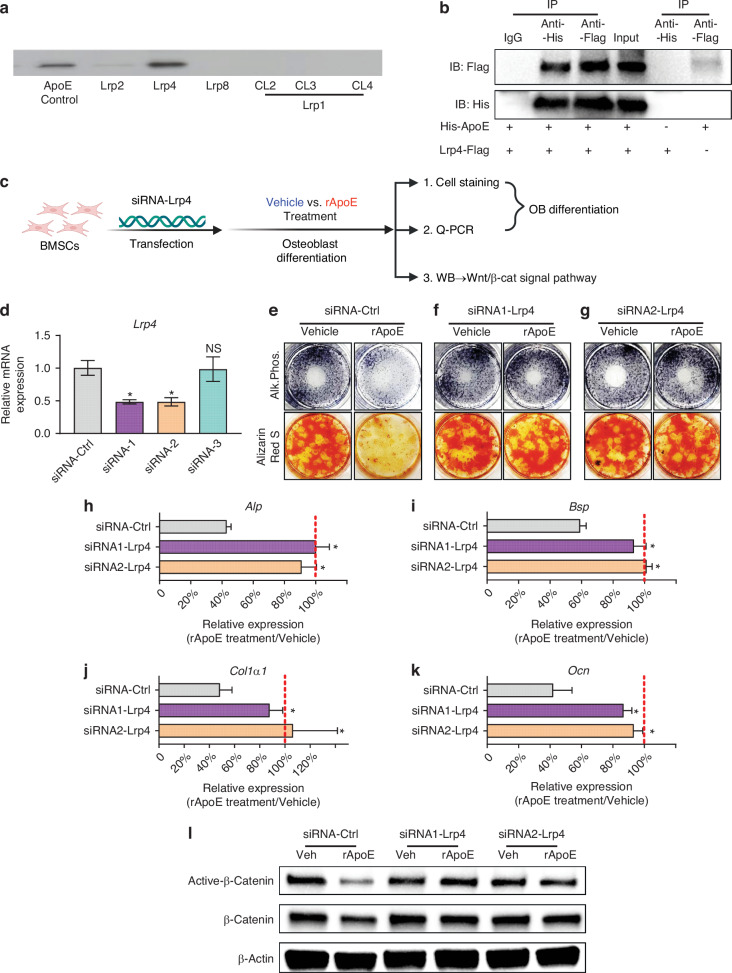
Lrp4 is the osteoblast cell-surface receptor for ApoE. **a** Potential ApoE binding partners were blotted using slot blot and probed with ApoE. ApoE-binding was visualized using HRP-conjugated anti-ApoE antibody. **b** BMSCs were transfected with plasmid, inducing expression of Flag-tag Lrp4. Cells were then treated with His-labelled ApoE and lysates were immunoprecipitated using anti-Flag and/or anti-His antibodies to confirm ApoE-Lrp4 binding. **c** Schematic diagram depicting siRNA-based knockdown of Lrp4 in BMSCs from 24-month-old C57BL/6 mice. **d** Confirmation of *Lrp4* gene knockdown in siRNA1-Lrp4 and siRNA2-Lrp4, but not siRNA-Ctrl. **e**–**g** Cells were washed, fixed, and stained for Alkaline Phosphatase (Alk. Phos.) or mineral (Alizarin Red) to assess osteoblast differentiation. **h**–**k** Relative expression of osteogenic transcripts (*Alp*, *Bsp*, *Col1a1*, and *Ocn*) was measured in all culture conditions. Transcript expression in rApoE-treated cultures was related transcript expression of vehicle-treated cultures and plotted as siRNA-Ctrl vs siRNA1-Lrp4 vs siRNA2-Lrp4. **l** Western blot analysis confirmed Lrp4 was required for ApoE-based modulation of β-catenin. Data are presented as mean ± 95% confidence interval. *P < 0.05

ApoE cell-surface receptors are members of the low-density lipoprotein receptor (LDLR) family. The core members of the LDLR family expressed on osteoblasts include LDLR-related protein 1(LRP1), LRP2 (aka megalin), LRP4 (MEDF7), and LRP8 (aka ApoE receptor-2, ApoER2).^13^ To identify the osteoblast cell-surface receptor through which ApoE inhibits Wnt/β-catenin signaling and osteoblast differentiation, we used a slot blot apparatus to interrogate ApoE binding to Lrp2, Lrp4, Lrp8, and clusters 2, 3, and 4 of Lrp1. We found that Lrp4 bound ApoE with the highest abundance (Fig. 4a). Lrp4 is an evolutionarily conserved transmembrane protein in LDL receptor family. To further investigate whether Lrp4 from BMSCs can bind to ApoE, we overexpressed Flag-tagged Lrp4 in BMSCs and collected cell lysate to treat it with His-tagged ApoE. We then studied Lrp4-ApoE binding in BMSC lysates using co-immunoprecipitation. In lysates containing both Flag-Lrp4 and His-ApoE, immunoprecipitation of one protein also sequestered the other (Fig. 4b). Collectively these findings confirm ApoE binds Lrp4.

Next, we used siRNA to decrease the expression of Lrp4 in BMSCs to determine whether ApoE signals through Lrp4 to inhibit osteoblast differentiation (Fig. 4c). RT-PCR results demonstrated that our 2 different siRNAs, siRNA1-Lrp4 (siRNA165574-Lrp4) and siRNA2-Lrp4 (siRNA-94437-Lrp4), targeted Lrp4 and successfully inhibited *Lrp4* expression by approximately 50% relative to the control siRNA-Ctrl (Fig. 4d). In siRNA-Ctrl cultures, ApoE treatment decreased the amount of Alkaline Phosphate and Alizarin Red staining. Importantly, knockdown of Lrp4 by siRNA1-Lrp4 or siRNA2-Lrp4 protected cultures from ApoE’s inhibitory effect as staining of cultures was unaffected by subsequent ApoE treatment (Fig. 4e–g). Furthermore, osteogenic transcripts (*Alp*, *Bsp*, *Col1a1*, and *Ocn*) were measured and normalized to siRNA-Ctrl. All four osteogenic transcripts were higher in siRNA1-Lrp4 and siRNA2-Lrp4 cultures than in siRNA-Ctrl (Fig. 4h–k). Finally, we tested the ability of ApoE treatment to modulate β-catenin levels in the absence of Lrp4. Active β-catenin levels were unchanged by ApoE treatment in siRNA1-Lrp4 and siRNA2-Lrp4 cultures while active β-catenin levels were decreased by ApoE-treatment in siRNA-Ctrl cultures, as demonstrated by Western blot analysis (Fig. 4l).

Collectively, these findings demonstrate that Lrp4 serves as the cell-surface receptor through which ApoE inhibits β-catenin signaling and osteoblast differentiation.

### Human osteoblast differentiation is inhibited by ApoE though the Lrp4-Wnt/β-catenin axis

**Fig. 5:**
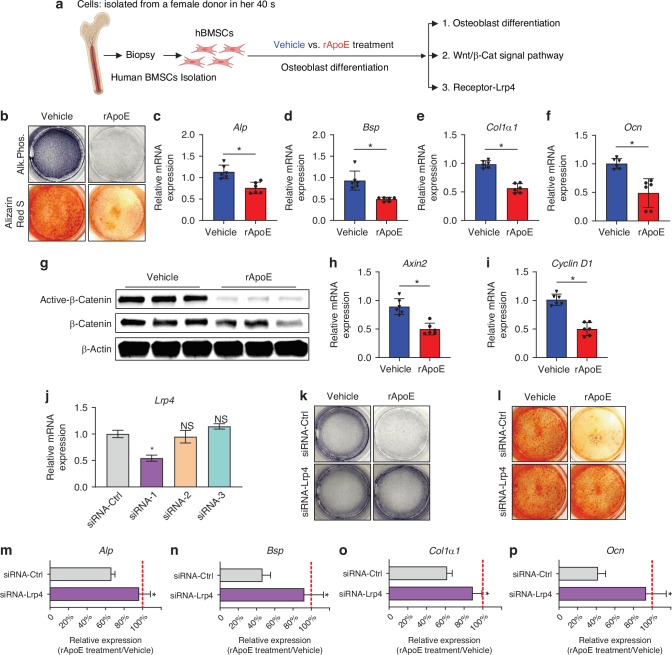
Human osteoblast differentiation is inhibited by ApoE through Lrp4-Wnt/β-catenin signaling. **a** Schematic diagram, human BMSCs were isolated from bone marrow, cultured, and differentiated in osteogenic media with vehicle or rApoE. **b** Cells were washed, fixed, and stained for Alkaline Phosphatase or mineral (Alizarin Red) to assess osteoblast differentiation. **c**–**f** Osteogenic transcripts (*Alp*, *Bsp*, *Col1*, and *Ocn*) were measured using RT-PCR (*n* = 6). **g** Western blot analysis of active-${\rm{\beta }}$-catenin and total ${\rm{\beta }}$-catenin protein levels in vehicle-treated and rApoE-treated groups. **h**, **i** Expression level of$\,{\rm{\beta }}$-catenin target genes *Axin2* and *Cyclin D1* (*n* = 6). **j** Confirmation of *Lrp4* gene knockdown in siRNA1-Lrp4. **k**, **l** Cells were washed, fixed, and stained for Alkaline Phosphatase (Alk. Phos.) or mineral (Alizarin Red) to assess osteoblast differentiation. **m**–**p** Relative expression of osteogenic transcripts (*Alp*, *Bsp*, *Col1a1*, and *Ocn*) was measured in all culture conditions. Transcript expression in rApoE-treated cultures was related transcript expression of vehicle-treated cultures and plotted as siRNA-Ctrl vs siRNA-Lrp4. Data are presented as mean ± 95% confidence interval. *P < 0.05

Thus far, we have used mouse models to demonstrate that circulating ApoE increases with age which impairs osteoblast differentiation by inhibiting the Wnt/β-catenin pathway through Lrp4-receptor signaling. As it does in mice, circulating ApoE increases with aging in human patients.^10^ We isolated human BMSCs (hBMSCs) from human bone marrow biopsies to determine whether ApoE can affect human osteoblast differentiation in a similar fashion as we see in mice. We used tissue culture techniques to assess 1) ApoE-dependent changes in osteoblast differentiation, 2) ApoE-dependent changes in Wnt/β-catenin signaling, and 3) the reliance on Lrp4 for ApoE-dependent changes (Fig. 5a).

First, hBMSCs were differentiated in osteogenic media in the presence of vehicle or rApoE. Alkaline Phosphate and Alizarin Red staining demonstrated that ApoE treatment decreased osteogenic activity and decreased matrix mineralization in these cells (Fig. 5b). Likewise, RT-PCR revealed that the expression level of osteoblast differentiation markers (*Alp*, *Bsp*, *Col1a1*, and *Ocn*) was significantly reduced in response to ApoE-treatment (Fig. 5c–f).

Next, we assessed changes in Wnt/β-catenin signaling in response to ApoE treatment. Western blot analysis of protein lysates demonstrated that ApoE treatment of hBMSCs decreased active and total β-catenin levels in osteogenic cultures (Fig. 5g). Furthermore, RT-PCR analysis of transcripts showed that ApoE treatment significantly decreased expression levels of β-catenin target genes *Axin2* and *Cyclin D1* (Fig. 5h, i).

Lastly, we used our siRNA system to investigate the role of Lrp4 in ApoE-induced inhibition of osteoblast differentiation. hBMSCs were cultured and transfected with either siRNA-Ctrl, siRNA1-Lrp4, siRNA2-Lrp4, or siRNA-3-Lrp4. siRNA1-Lrp4 successfully lowered *Lrp4* levels (Fig. 5j). Fresh hBMSCs were then cultured and transfected with either siRNA-Ctrl or siRNA1-Lrp4. Cultures were maintained in osteogenic media with vehicle or rApoE. ApoE treatment had no effect on siRNA1-Lrp4-treated cultures but decreased Alkaline Phosphatase and Alizarin Red staining of siRNA-Ctrl-treated cultures (Fig. 5k, l). Next, hBMSCs treated with siRNA-Ctrl or siRNA1-Lrp4 were cultured in osteogenic media containing ApoE. The level of osteogenic transcripts was compared between the two groups to assess the impact of Lrp4 knockdown in ApoE-based inhibition of osteoblast differentiation. In siRNA1-Lrp4 cultures, knockdown of Lrp4 rendered the cells insensitive to ApoE treatment (Fig. 5m–p).

Collectively, these data demonstrate that similar to the mechanism we identified in mouse models, ApoE inhibits human BMSC-to-osteoblast differentiation by binding to Lrp4 and inhibiting Wnt/β-catenin signaling.

### Neutralizing circulating ApoE improves bone fracture healing in aged mice

**Fig. 6:**
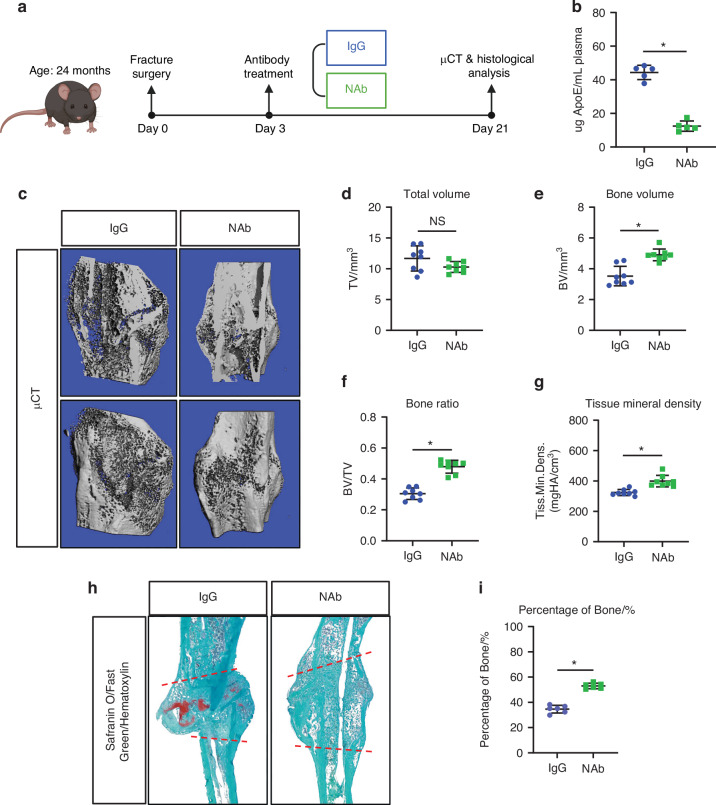
ApoE-neutralizing antibody improves aged bone healing. **a** Schematic diagram of fractured 24-month-old mice treated with ApoE-neutralizing antibody and assessed for fracture healing. **b** Mice were injected with IgG or NAb 3 days post injury and blood was collected 7 days post-injection and assessed for ApoE concentration (n = 5). **c** Micro-CT analysis of 21-day fracture calluses was used to determine (**d**) total volume (TV), (**e**) bone volume (BV), (**f**) bone deposition (BV/TV), and (**g**) tissue mineral density (*n* = 8). **h** Safranin O/fast green staining was used to stain the fracture calluses and (**i**) histomorphometric analysis was used to quantify the amount of bone within fracture calluses (*n* = 6). Data are presented as mean ± 95% confidence interval. *P < 0.05

Next, we tested the ability of an ApoE-neutralizing antibody to transiently lower circulating ApoE and counteract the negative effects on bone regeneration. To this end, we tested the ability of an ApoE-neutralizing antibody (NAb, HJ6.3) to improve aged fracture healing (Fig. 6a).

24-month-old mice were treated with IgG control or NAb (10 mg/kg, IP). Plasma collected 7 days post-injection showed a 75% decrease in circulating ApoE concentrations (Fig. 6b). Liver panels and lipid levels were performed using HPLC/MS: total cholesterol, HDL, LDL, triglycerides, albumin, AST, and ALT were all measured in samples from IgG control and NAb-treated mice. Cholesterol, HDL, and LDL were all unchanged, indicated no significant changes in plasma lipid levels (Fig. S6). ALT was also unchanged however; triglycerides were slightly increased in NAb samples while albumin and AST were slightly decreased in NAb samples (Fig. S6). Therefore, while there were slight changes in triglycerides, albumin, and AST, NAb treatment was successful in lowering circulating ApoE levels.

Next, 24-month-old mice underwent tibial fracture surgery and were treated with NAb or IgG 3 days after injury. Fracture calluses were collected 21 days post-fracture and assessed using micro-CT and histological techniques). Micro-CT analysis of fracture calluses demonstrated that NAb treatment did not change the total volume; however, bone volume, bone ratio, and tissue mineral density were significantly increased (Fig. 6c–g). Moreover, histomorphometry of Safranin O/fast green/hematoxylin stained sections revealed that fracture callus of the NAb-treated group contained approximately 50% more bone tissue than did the IgG-treated control group (Fig. 6h, i). Furthermore, cartilage formation and osteoclast activity was unaffected 14-day post fracture (Fig. S7) however, osteoblast numbers were significantly increased in 21-day NAb-treated fracture calluses while cartilage template was decreased, and osteoclast activity was unchanged (Fig. S8).

Importantly, circulating ApoE levels and fracture healing was unaffected by NAb treatment of young mice (Fig. S9). Collectively, these data demonstrate that treatment with ApoE-neutralizing antibody lowers circulating ApoE levels in aged mice which significantly improves bone fracture healing.

## Discussion

In this study we identified the mechanism of action by which hepatic ApoE inhibits fracture healing and identified a translatable non-invasive therapeutic intervention to improve aged bone repair. We nulled hepatic ApoE expression by using Alb-Cre;ApoE^fl/fl^ (ΔApoE) mice - this decreased levels of circulating ApoE and increased bone deposition and tissue mineral density within the fracture callus. Using tissue culture models, we found ApoE inhibits BMSC-to-osteoblast differentiation and activity by binding to the cell-surface receptor Lrp4 and inhibiting Wnt/β-catenin signaling. Moreover, the same mechanism of action was identified during ApoE-induced inhibition of human BMSCs. Finally, aged wildtype mice underwent tibial fracture surgery and were treated with NAb 3 days post-injury which decreased levels of circulating ApoE and significantly improved fracture healing.

In our previously published study, we demonstrated that circulating ApoE levels increase with age in patients and in mice and that by using hepatotropic AAV-siRNA-ApoE we decreased circulating ApoE levels and increased bone deposition and mechanical stability of healed tissue.^10^ However, the potential negative impact on a patient’s cardiovascular health resulting from the permanent lowering of ApoE precludes this therapeutic strategy.^14–16^ Therefore, in the current study we aimed to use a neutralizing antibody against ApoE which would be cleared from the body by immune cells. This would function as a transient intervention, lowering circulating ApoE upon treatment with gradual cessation, minimizing any potential effects to liver or lipid health. A similar approach has recently been used to successfully sequester ApoE in the brain leading to prevention of plaque formation in mouse models of Alzheimer’s disease.^17–19^ Our use of this antibody decreased levels of circulating ApoE and improved fracture healing in aged mice. Importantly, off-target effects appear to be minimized as liver panels and lipid levels displayed only minor changes in response to NAb treatment.

Our proposed therapeutic strategy bears a striking resemblance to the FDA approved anti-sclerostin antibodies used to treat osteoporosis.^20^ Sclerostin is a circulating protein that inhibits Wnt binding to Lrp5/6.^21^ Anti-sclerostin antibodies bind and neutralize sclerostin allowing Wnt binding to Lrp5/6 and sequestration of the β-catenin destruction complex. This allows β-catenin to localize into the nucleus and induce transcription of Wnt/β-catenin signaling targets which increases osteoblast differentiation and activity. In our case, it is important to mention that our ΔApoE mouse model did not display a developmental bone phenotype. This indicates that circulating ApoE may not play a critical role in bone homeostasis but rather may only play a role in aged fracture healing. Furthermore, NAb-treatment of young mice did not affect ApoE levels nor fracture healing outcomes. Collectively these data indicate a potential threshold that serum ApoE levels must surpass to inhibit osteoblast biology. Indeed, in tissue culture models we identified ApoE to inhibit osteoblastic differentiation passed a specific threshold in cultures from mouse and human cells (Fig. S10). Thus, it is possible that ApoE only inhibits osteoblast differentiation during times of rapid bone formation, like during fracture healing and not during homeostasis, like during aging. However, future work investigating the efficacy of using ApoE-neutralizing antibodies to treat osteoporosis may shed light on this anomaly.

ApoE inhibits osteoblast differentiation and activity. This is an effect previously reported by us and others ^22,23^ however, our discovery and confirmation that ApoE inhibition of osteoblasts works through Lrp-4 and Wnt/B-catenin signaling is the first mechanistic insight provided. Lrp4 and the Wnt/β-catenin pathway have been well considered in bone biology. Wnt/β-catenin is an obligate pathway, known to be required for normal osteoblast differentiation and robust bone healing.^24–26^ More recently, Lrp4-receptor binding has been demonstrated to inhibit osteoblast activity through inhibition of the Wnt/β-catenin pathway.^27–29^ Our study demonstrates that in both mice and humans, ApoE binds the Lrp4-receptor on osteoblast cell surface and is responsible for downregulation of Wnt/β-catenin signaling—as shown by decreased active β-catenin levels and decreased transcript levels of downstream targets in the Wnt/β -catenin pathway. Most importantly, these mechanistic findings were validated in human BMSC cultures.

Along with the Wnt/β-catenin pathway, our RNA sequencing analysis identified Hippo signaling to be altered in response to ApoE treatment of osteoblast cultures. However, deeper analysis using Taz-floxed/Yap-floxed cell culture models revealed that Hippo signaling was not required for ApoE’s inhibitory activity. In our study Hippo signaling was likely implicated due to cross-talk with Wnt/β-catenin signaling which stems from Yap/Taz being a member of the β-catenin destruction complex and not due to direct ApoE signaling.^30^

In summary, we determined that hepatic ApoE inhibits aged bone fracture healing by decreasing bone deposition within the fracture callus. Furthermore, we identified the underlying mechanism of action: circulating ApoE binds to Lrp4 to inhibit Wnt/β-catenin signaling and to inhibit osteoblast differentiation and activity. This mechanism was validated in human BMSCs. An ApoE-neutralizing antibody was administered systemically, post-injury, and improved bone fracture healing in an aged mouse model. Our work sheds new light on ApoE serving as a therapeutic target to improve bone healing in aged patients.

## Materials and methods

### Study design

The aim of this study was to identify the molecular mechanism by which ApoE impairs aged bone fracture healing and to use this knowledge to develop a translatable therapeutic intervention that improves aged bone fracture healing. To complete these goals, we used a combination of mouse tibial fracture models and mouse or human cell culture models. First, as the liver is the primary source of circulating ApoE, we deleted hepatic expression of ApoE using Albumin-Cre;ApoE^fl/fl^ (ΔApoE) mice. In aged mice circulating ApoE was decreased and bone fracture healing was improved. Next, we used tissue culture models to investigate the effect of ApoE on osteoblast differentiation. ApoE inhibited osteoblast differentiation and RNA sequencing indicated the Wnt/β-catenin and Hippo pathways to be the most differentially regulated (inhibited) pathways by ApoE treatment. RNA sequencing findings were confirmed by transcript and protein analysis. Next BMSCs from transgenic mice in which β-catenin could not be modulated or Yap/Taz was knocked out were differentiated to osteoblasts in the presence of vehicle or ApoE. This work confirmed ApoE’s inhibitory activity functions through the Wnt/β-catenin pathway. Next, we investigated known ApoE receptors that are expressed on the osteoblast surface. Lrp4 displayed preferential binding to ApoE. Indeed, loss of Lrp4 expression on osteoblasts nulled ApoE’s inhibitory activity. These mechanistic studies were then repeated using human bone marrow stromal cells. Human cell cultures recapitulated our findings in mouse cell cultures. Finally, we used an ApoE-neutralizing antibody in aged, wildtype mice which lowered circulating ApoE and improved aged fracture healing.

### Mice

All animal protocols were approved by the Duke Institutional and Animal Care and Use Committee. All mice were used at either 4-months (young) or 24-months (old) of age. Wildtype mice (C57BL/6 J, stock 000664), ApoE^fl/fl^ mice (B6.129S6-Apoetm1.1Mae/MazzJ, stock 028530), Albumin-Cre mice (B6.Cg-Speer6-ps1Tg(Alb-cre)21Mgn/J, stock 003574), and Yap/Taz double floxed mice (Wwtr1tm1.2Hmc Yap1tm1.2Hmc/WranJ,stock 030532) were purchased from the Jackson laboratory. β-catenin stabilized mice bearing loxP sites flanking exon 3 in which Cre-recombinase-mediated excision leads to a stabilized form of β-catenin protein ^8,31^ were a generous gift from Dr. BA Alman. To achieve liver-specific deletion of ApoE, Albumin-Cre mice were crossed with ApoE^fl/fl^ mice to generate Albumin-Cre^+/−^;ApoE^fl/fl^ (ApoE^Alb^ mice) and Albumin-Cre^−/−^;ApoE^fl/fl^ mice (ApoE^fl/fl^ mice).

### Tibial fracture surgery

Tibial fracture surgery was performed as previously described.^32,33^ Briefly, mice were anesthetized using 2% isoflurane and buprenorphine-SR (0.5 mg/kg) was provided for analgesia. The surgical site proximal to the knee was shaved and sterilized. Following an incision, a hole was drilled into the tibial plateau, and a 0.7-mm stainless steel pin was carefully inserted into the medullary cavity and cut flush with the tibial plateau. A tibial fracture was induced at the midshaft using blunt scissors, and the incision was subsequently closed using wound clips. The animals were monitored throughout the surgery and during recovery.

### Drillhole surgery

Mice were anesthetized using 2% isoflurane and buprenorphine-SR (0.5 mg/kg) was provided for analgesia. The surgical site distal to the knee was shaved and sterilized. Following an incision, a hole was drilled into one cortex of the tibial using a 25 G needle. The incision was subsequently closed using wound clips. The animals were monitored throughout the surgery and during recovery.

### Micro-CT analysis

Intact tibiae containing the fracture calluses or drill-hole defects were dissected and fixed in 10% Zn-formalin at room temperature for 5 days. Following fixation, tibiae were scanned using a Scanco vivaCT 80 (Scanco Medical) at a resolution of 8 μm. Assessment of the radiographic slices within the fracture callus was conducted to identify the midpoint of the fracture site. Fracture calluses were analyzed 1 mm proximal and 1 mm distal from the midpoint. Drill-hole defects were assessed in their entirety. Fracture calluses were assessed for total volume (TV), bone volume (BV), percentage of BV per TV, and bone mineral density. Drill-hole defects were assessed for percentage of BV per TV.

### Histology and immunohistochemistry

After radiographic imaging, fixed fracture calluses were decalcified in 14% EDTA (pH 7.40), cleared of EDTA, embedded in paraffin, and sectioned at 5 µm. Safranin O/fast green/hematoxylin staining was performed to visualize fracture calluses. The sections were mounted with Permount mounting medium (Fisher Chemical, SP15-100). Images were acquired using an Olympus IX70 microscope and quantified using Image J software. For histomorphometry analysis a minimum of 5 sections was analyzed and the results were presented as relative quantities with respect to the area of the fracture callus.

### ApoE-neutralizing antibody treatment

24-month old male mice underwent tibial fracture surgery and were treated with IgG (control) or HJ6.3 (NAb, anti-ApoE antibody) at 10 mg/kg, 3 days after fracture injury. 21 days after fracture surgery, mice were euthanized and assessed for fracture healing. ApoE levels were measured from plasma using ELISA (Abcam, ab215086) as per manufacturers’ instructions.

### Liver panel and lipid analysis

Blood was collected from IgG and NAb-treated mice 7 days post injection and analyzed using HPLC/MS for cholesterol, HDL, LDL, triglycerides, ALT, AST, and albumin using the Duke Molecular Measures Core.

### Mouse bone marrow stromal cell culture

Primary bone marrow stromal cells were isolated from the femurs and tibiae of unfractured 4- or 24-month-old mice as previously described.^34,35^ Briefly, soft tissue was dissected from long bones and bone marrow was flushed and dissociated by passing through an 18 G needle. The collected cells were passed through a 70 nm nylon mesh (BD Falcon) and plated at a density of (500 × 10^3^)/cm^2^ in plating medium (α-MEM, 10%FBS, 100 U/mL penicillin/streptomycin). BMSCs were differentiated to osteoblasts in osteogenic medium (α-MEM, 10%FBS, 100 U/mL penicillin/streptomycin, 30 μmol/L ascorbic acid, 10 nmol/L dexamethasone, 8 mmol/L sodium phosphate). ApoE treatment (100 ng/mL of recombinant ApoE) was carried out beginning two days after the addition of osteogenic media and throughout the duration of differentiation. After 15 days, wells were washed with PBS and fixed using 10% formalin. Wells were then stained for Alkaline Phosphate (Millipore) or for mineral deposition (Alizarin Red S) (Sigma).

In a subset of stabilized-β-catenin^fl/fl^ and Yap/Taz^fl/fl^ BMSC cultures, Cre-recombinase expression was induced using adenovirus. Wells were treated with adenovirus encoding either Cre recombinase (Ad-Cre, multiplicity of infection =100; Vector Biolabs) or GFP (Ad-GFP, multiplicity of infection =100; Vector Biolabs) for 4 days, immediately prior to using osteogenic media with or without recombinant ApoE.

### siRNA transfection

*Lrp4-*targeting siRNAs and negative control siRNA were purchased from Thermo Fisher Scientific. Bone marrow stromal cells (BMSCs) were seeded in culture plates one day prior to transfection. Transfections were performed using Lipofectamine™ 2000 (Thermo Fisher Scientific) according to the manufacturer’s protocol. After 24 h of transfection, cells were induced to undergo osteogenic differentiation using osteoblast differentiation medium. To maintain knockdown efficiency during the differentiation period, siRNA transfection was repeated every three days. At the end of the differentiation protocol, cells were harvested for downstream analyses.

### Human bone marrow stromal cell culture

Human bone marrow stromal cell culture – Primary human bone marrow stromal cells (hBMSCs) were isolated from bone marrow biopsies obtained from a female donor in her 40 s, as previously described.^36^ Human bone marrow aspirates were cultured in growth medium consisting of α-MEM supplemented with 10% fetal calf serum, 100 U/mL penicillin/streptomycin, and 10 ng/mL basic fibroblast growth factor (bFGF). After 2 days, non-adherent cells were removed by washing, and the adherent cells were expanded.

At subconfluence, hBMSCs were trypsinized for cell passaging. Cells from passages 3 to 6 were used for experiments. For osteoblast differentiation, the same methods were applied as those used for mouse BMSCs.

### RT-PCR

Total RNA was extracted from the cultured cells using TRIzol Reagent (Invitrogen) following the manufacturer’s instructions. cDNA was synthesized using a reverse transcription kit (Thermo Fisher Scientific) according to the manufacturer’s instructions. Realtime PCR was performed using the SYBR Green PCR Master Mix (Thermo Fisher Scientific). Samples were investigated using a ViiA 7 RT-PCR System and compared to the transcript of GAPDH as a housekeeping control. Primer sequences are provided in Supplementary Table 1.

### RNA-seq and data processing

After eight days of osteoblast differentiation, BMSCs treated with vehicle or rApoE were collected. Total RNA was isolated using TRIzol Reagent (Invitrogen) following the manufacturer’s instructions. Subsequently, complementary DNA (cDNA) library preparation and sequencing were performed using Illumina’s standard protocol. RNA-seq reads were mapped to the GRCm39 mouse genome (Ensembl) using the STAR RNA-seq alignment tool.^37^ Gene counts were normalized, and differential expression analysis was performed using the DESeq2 package^38^ from Bioconductor, implemented in the R programming environment. Differentially expressed genes were identified using DESeq2 with a cut-off Benjamini-Hochberg-adjusted *P* value < 0.05 and |log_2_FC | > 1.5. Data is available as GEO309814.

### Western blot analysis

After 12 days of osteoblast differentiation, BMSCs treated with vehicle or rApoE were washed three times with PBS and lysed with RIPA lysis buffer. The extracted proteins were then separated using SDS-PAGE gels (Bio-Rad) and transferred to a PVDF membrane (Bio-Rad). The PVDF membrane was blocked with 5% BSA in PBS, incubated with specific antibody targeting the protein of interest, and the appropriate secondary antibody. The blot was washed with TBST (tween-20 0.1%) after each antibody incubation and the final blot was visualized using ECL (Cytiva). Quantitative data were analyzed by Image Lab software.

### Slot blot

A slot blot apparatus (Bio-Rad) was used to perform a dot blot assay. Nitrocellulose membrane was inserted into the slot blot manifold and 1 µg of each protein was blotted into adjacent matrices. Vacuum pressure was applied and the member was blocked in 5% milk in TBST. Membrane was then blotted in 1 µg/mL rApoE solution in TBS, washed with 5% milk in TBST, and incubated with HRP-conjugated anti-ApoE antibody (Abcam, ab195855). The final blot was visualized using ECL (Cytiva).

### Co-Immunoprecipitation (Co-IP) Assay

Mouse BMSCs transfected with Flag-Lrp4/pCDNA3.1 plasmid (Addgene) were lysed in RIPA lysis buffer (Millipore) supplemented with a protease inhibitor cocktail (Roche) at 4 °C for 30 min. The lysates were then centrifuged at 12 000 × *g* for 15 min, and the resulting supernatant was incubated with His-ApoE recombinant protein (ACROBiosystems) at 4 °C for 1 h. Following this, the supernatant was incubated with either an anti-Flag antibody or an anti-His antibody (Cell Signaling Technology) overnight at 4 °C. The protein-antibody complexes were then incubated with Protein A magnetic beads (Thermo Fisher Scientific) with rotation at 4 °C for 2 h. Finally, the protein samples were collected for subsequent western blot analysis.

### Immunostaining

Cultures were washed three times with PBS and fixed in 10% Zn-formalin at room temperature for 15 min. Cells were permeabilized in 0.1% Triton X-100 for 10 min at room temperature and washed in PBS. Cells were then blocked in 1% BSA for 30 min at room temperature and incubated overnight at 4 °C with primary antibodies in blocking buffer. Cells were washed three times with PBS and incubated with flurophore-conjugated secondary antibodies in the blocking buffer for 1 h at room temperature followed by washing in PBS and stained with DAPI for 5 min at room temperature. Finally, Images were acquired using an Olympus IX70 microscope.

### Statistical analysis

All results are presented as the mean ± SD. Statistical analysis was performed using GraphPad PRISM 9 (GraphPad software). For comparison between two groups, two-tailed, unpaired Student’s *t* test was applied. One-way ANOVA with Tukery’s post hoc multiple comparisons was used when the data involves multiple group comparison. Statistical significance was assigned to *P* values less than 0.05. Each experiment was performed with three technical replicates.

## Supplementary information

Supplemental Material
