# Supplementary material for: Neutralizing hepatic apolipoprotein E enhances aged bone fracture healing
Source: Bone Res. 2026 Jan 22;14:13. doi: 10.1038/s41413-025-00489-y (PMC12827301; doi:10.1038/s41413-025-00489-y)
Supplement: Supplementary file 1 — Supplemental Material [file 41413_2025_489_MOESM1_ESM.docx]

**
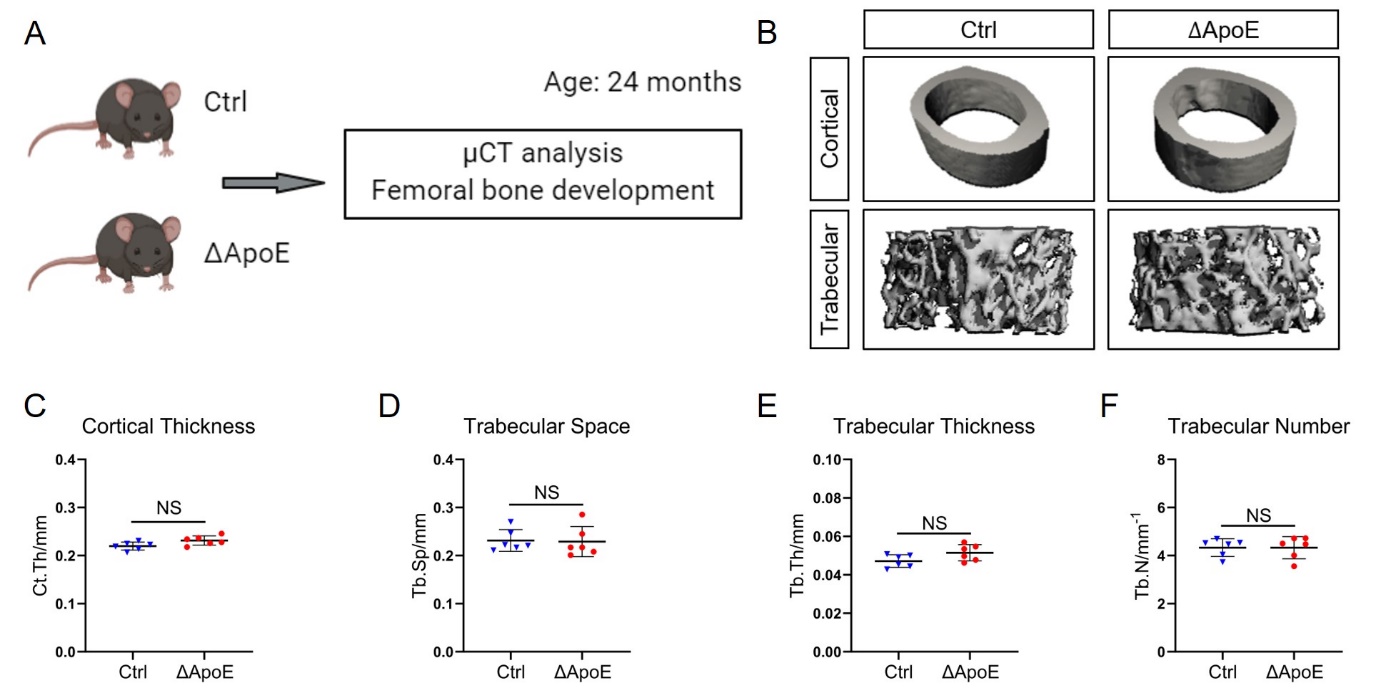
**

**Supplemental Figure 1 – Loss of hepatic ApoE expression does not alter homeostatic bone metrics.** (A) Schematic diagram of radiographic analysis of femurs from 24-month-old control (ApoE^fl/fl^, Ctrl) mice and liver-specific ApoE knockout (ApoE^Alb^, ΔApoE) mice. Micro-CT analysis was used to (B) produce images of cortical and trabecular bone and determine (C) cortical thickness, (D) trabecular space, (E) trabecular thickness, and (F) trabecular number. (Ctrl, n=6; ΔApoE, n=6); Data are presented as mean ± 95% confidence interval; * p <0.05.

**Supplemental Figure 2 – Loss of hepatic ApoE expression increases osteoblast numbers but does not change osteoclast numbers.** 24-month old control (ApoE^fl/fl^, Ctrl) mice and liver-specific ApoE knockout (ApoE^Alb^, ΔApoE) mice underwent tibial fracture surgery and fracture calluses were assessed 21-days post injury. A) Cartilage was quantified using histomorphometry of Safranin O/fast green staining and displayed as percent cartilage of whole fracture callus. B) Osteoblast numbers were determined using osteocalcin (OCN) staining and displayed as osteoblast surface per bone surface (Ob.S/BS), C) Osteoclast numbers were determined using tartrate resistant acid phosphatase (TRAP) staining and displayed as osteoclast surface per bone surface (Oc.S/BS). (Ctrl, n=6; ΔApoE, n=6). Data are presented as mean ± 95% confidence interval. * p <0.05.

**Supplemental Figure 3 – Loss of hepatic ApoE expression increases osteoblast numbers but does not change osteoclast numbers within the drillhole model.** 24-month old control (ApoE^fl/fl^, Ctrl) mice and liver-specific ApoE knockout (ApoE^Alb^, ΔApoE) mice underwent drillhole defect. Healing was assessed 14 days after surgery. A) Osteoclast numbers were determined using tartrate resistant acid phosphatase (TRAP) staining and displayed as osteoclast surface per bone surface (Oc.S/BS). C) Osteoblast numbers were determined using osteocalcin (OCN) staining and displayed as osteoblast surface per bone surface (Ob.S/BS). (Ctrl, n=6; ΔApoE, n=6). Data are presented as mean ± 95% confidence interval. * p <0.05.

**Supplemental Figure 4 – ApoE impairs osteoblast differentiation of young bone marrow stromal cells.** (A) Schematic diagram of BMSCs isolated from 4-month old mice, cultured, and subsequently differentiated in osteogenic media containing vehicle or rApoE (100ng/mL). (B-D) Transcripts for osteogenic genes (*Alp*, *Bsp*, and *Ocn*) were measured using RT-PCR. (n=6). Data are presented as mean ± 95% confidence interval. * p <0.05.


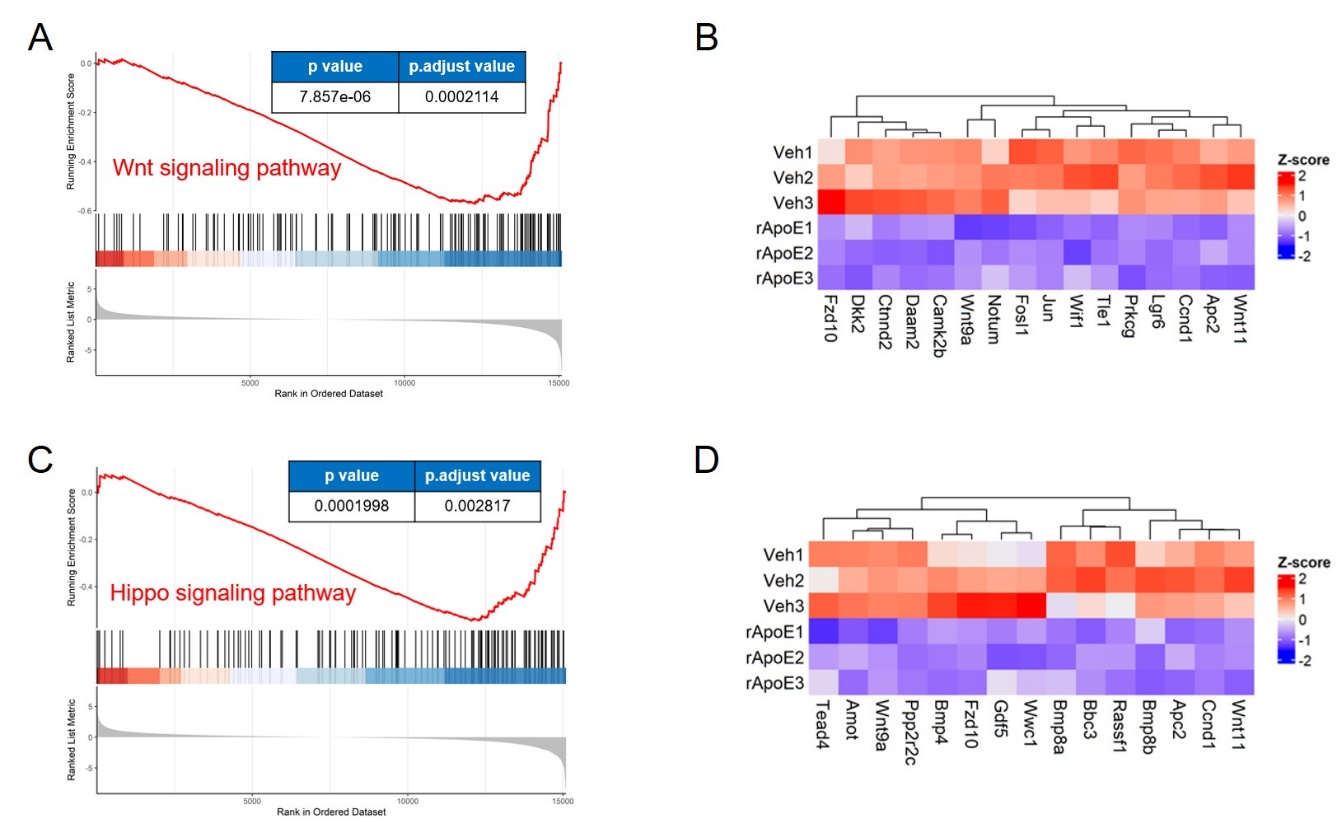


**Supplemental Figure 5 – RNA sequencing analysis identifies Wnt/β-catenin and Hippo signaling to be inhibited after ApoE-treatment of differentiating osteoblasts.** (A) Gene set enrichment analysis (GSEA) showed that the Wnt signaling pathway is significantly enriched in rApoE-treated BMSCs during osteoblast differentiation compared to the vehicle-treated group. (B) Heatmap illustrated the relative mRNA expression levels of Wnt signaling-related genes in vehicle-treated group and rApoE-treated groups. (C) GSEA revealed that the Hippo signaling pathway is significantly enriched in rApoE-treated BMSCs during osteoblast differentiation. (D) Heatmap indicated the relative mRNA expression levels of Hippo signaling-related genes in vehicle-treated group and rApoE-treated groups.

**
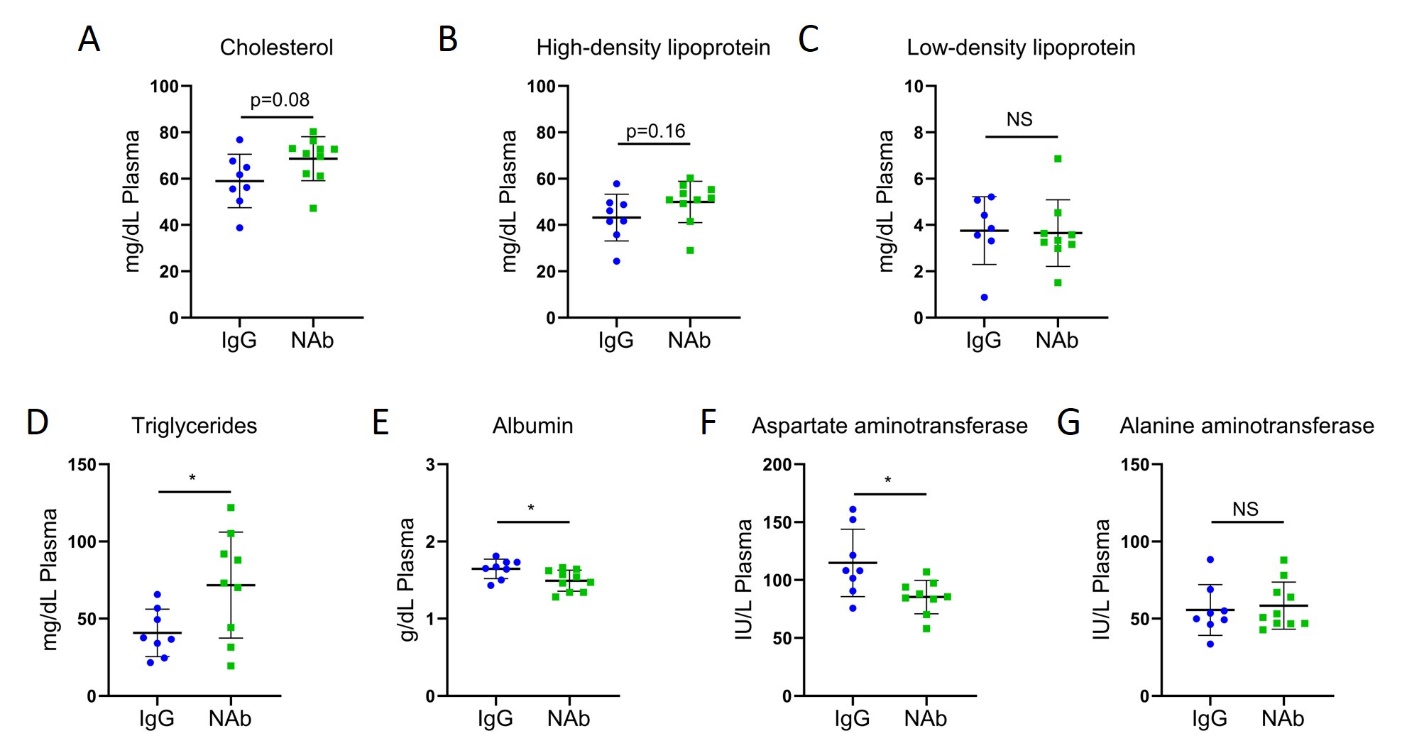
**

**Supplemental Figure 6 – Plasma analysis shows only slight changes in lipid and liver panels after NAb treatment.** 24-month-old mice were injected with ApoE neutralizing antibody and plasma samples were collected 7 days post-injection. HPLC/MS was used to quantify (A) total cholesterol, (B) HDL, (C) LDL, (D) triglycerides, (E) albumin, (F) AST, and (G) ALT. (IgG, n=8; NAb, n=9); Data are presented as mean ± 95% confidence interval; * p <0.05.

**Supplemental Figure 7 – ApoE-neutralizing antibody does not alter early stages of aged fracture healing.** Fractured 24-month old mice were treated with IgG or NAb 3 days after surgery. Fracture calluses were assessed 14-days post injury. A) Histomorphometry of Safranin O/fast green staining was used to assess cartilage and bone formation - B) as percent cartilage of whole fracture callus and C) as percent bone of whole fracture callus. D) Osteoclast numbers were determined using tartrate resistant acid phosphatase (TRAP) staining and E) displayed as osteoclast surface per bone surface (Oc.S/BS). (IgG, n=6; NAb, n=6) Data are presented as mean ± 95% confidence interval. * p <0.05.

**Supplemental Figure 8 – ApoE-neutralizing antibody increases osteoblast numbers but does not change osteoclast numbers.** Fractured 24-month old mice were treated with IgG or NAb 3 days after surgery. Fracture calluses were assessed 21-days post injury. A) Cartilage was quantified using histomorphometry of Safranin O/fast green staining and displayed as percent cartilage of whole fracture callus. B) Osteoclast numbers were determined using tartrate resistant acid phosphatase (TRAP) staining and displayed as osteoclast surface per bone surface (Oc.S/BS). C) Osteoblast numbers were determined using osteocalcin (OCN) staining and displayed as osteoblast surface per bone surface (Ob.S/BS). (Ctrl, n=6; ΔApoE, n=6). Data are presented as mean ± 95% confidence interval. * p <0.05.

**Supplemental Figure 9 – ApoE-neutralizing antibody does not affect fracture healing in young mice.** (A) Schematic diagram of fractured 4-month-old mice treated with ApoE-neutralizing antibody and assessed for fracture healing. (B) Mice were injected with IgG or NAb 3 days post injury and blood was collected 7 days post-injection and assessed for ApoE concentration. (C) Micro-CT analysis of 21-day fracture calluses was used to determine (D) total volume (TV), (E) bone volume (BV), (F) bone deposition (BV/TV), and (G) tissue mineral density. (IgG, n=7; NAb, n=8) Data are presented as mean ± 95% confidence interval. * p <0.05.

**Supplemental Figure 10 – Threshold at which ApoE exerts osteoblastic inhibitory activity.** BMSCs from 24-month old mice or a 40-year old human patient were cultured and differentiated in osteogenic media containing increasing amounts of recombinant ApoE. Transcripts for osteogenic genes (*Alp*, *Bsp*, and *Ocn*) were measured using RT-PCR and Col1a1 levels were measured using Western blot analysis. (n=4 for RT-PCR, n=3 for Western). Data are presented as mean ± 95% confidence interval.

**Supplemental Table 1 – List of RT-PCR primers.**

**
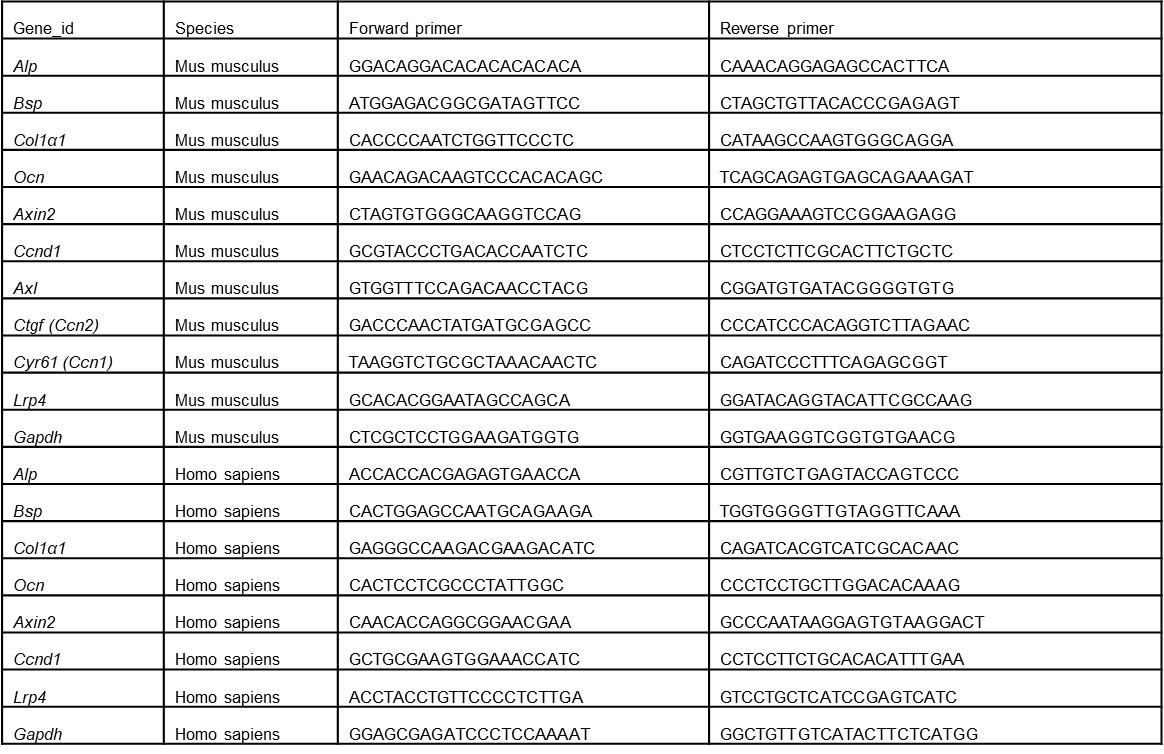
**
